# New “Wrinkle Method” for Intracorporeal Anterior Vaginal Wall Plication during Sacrocolpopexy

**DOI:** 10.3390/jcm10091822

**Published:** 2021-04-22

**Authors:** Sa Ra Lee, Ju Hee Kim, Sung Hoon Kim, Hee Dong Chae

**Affiliations:** Department of Obstetrics and Gynecology, Asan Medical Center, University of Ulsan College of Medicine, 88, Olympic-ro 43-gil, Songpa-gu, Seoul 05505, Korea; xjuheex@gmail.com (J.H.K.); kimsung@amc.seoul.kr (S.H.K.); hdchae@amc.seoul.kr (H.D.C.)

**Keywords:** erosion, pelvic organ prolapse, recurrence, sacrocolpopexy, wrinkle method

## Abstract

In this study, we introduce a new wrinkle method for intracorporeal anterior vaginal wall plication during sacrocolpopexy for pelvic organ prolapse (POP) aiming to decrease POP recurrence and postoperative vaginal wall mesh erosion. The wrinkle method was performed using robotic sacrocolpopexy (RSC) on 57 symptomatic POP patients. Sixty-six patients underwent conventional RSC before the development of the wrinkle method. Feasibility and perioperative outcomes were compared. The wrinkle method is not time consuming. The total operative time was shorter in the wrinkle group than in the non-wrinkle group; however, this was attributed to lower adhesiolysis in the wrinkle group. No differences were recorded in the mean estimated blood loss and complication rates between the two groups. In conclusion, although we were unable to confirm that the wrinkle method decreased POP recurrence and vaginal wall mesh erosion after RSC because of the short follow-up period, our preliminary findings are positive in terms of safety. Further long-term well designed randomized controlled trials are required to elucidate the benefits of the wrinkle method.

## 1. Introduction

Pelvic organ prolapse (POP) is a common condition in older women, with a prevalence of 41–50% in examination, and a probability of 12.6–20% to undergo surgery for POP before the age of 80 years [1,2,3,4,5]. Sacrocolpopexy (SC) is a common reconstructive procedure to correct apical POP, which suspends the anterior and posterior vaginal walls to the anterior longitudinal ligament (ALL) of the sacrum using a mesh or graft. Open SC has been the gold standard procedure for patients with apical POP, with long-term cure rates reaching 90% [6,7,8]. The use of minimally invasive SC (MISC), including laparoscopic SC (LSC) and robotic SC (RSC), has been increasing, and large clinical data indicate that LSC is replacing open SC as the gold standard procedure as it provides low recurrence rates and low comorbidity rates similar to those of open surgery [9,10,11].

Mesh-related morbidity, including vaginal mesh erosion, chronic pain, and reoperation, is a crucial issue. The mesh erosion rate after pelvic reconstructive surgeries has been reported to be much lower in the laparoscopic approach (2–8%) [12,13] than in the vaginal approach (10.3%) in a systematic review [14], leading to an FDA warning [12]. However, surgeons should consider decreasing the mesh erosion risk when using mesh in pelvic reconstructive surgery.

Risk factors associated with vaginal mesh erosion can be classified as mesh-related, patient-related, and surgery-related. Monofilament, macroporous, light-weighted, and soft mesh can decrease mesh erosion; however, smoking, the use of steroids, diabetes, premenopause, estrogen replacement therapy during postmenopause, and an advanced stage of POP can increase mesh erosion [15,16,17,18,19,20,21]. Concomitant hysterectomy, intra-abdominal adhesions, and postoperative pelvic hematoma, lack of surgical experience, and incorrect indication may increase the risk of vaginal mesh erosion after SC [22,23,24,25,26,27].

The recurrence rate of POP after various types of reconstructive surgeries is reported to be as high as 30% [28,29], which is very frustrating for both patients and surgeons, not only because of the need for reoperation but also because most POP patients are older women with high surgical risk [30,31]. In those cases, a pessary insertion can be an option for recurring POP management. However, since older women often experience difficulty in both daily removal and re-insertion of the pessary, they need to visit the outpatient gynecologic clinic for pessary change. Therefore, a pessary is not always a good option for many older women who may have mobility issues or disability. Therefore, the low SC recurrence rate (approximately 6% [32]) is important not only for relatively young, healthy, active POP patients but also for older POP patients. When recurrence occurs after SC, anterior compartment prolapse is most commonly involved [33,34,35]. In terms of determining an effective method of decreasing the recurrence of anterior compartment POP after SC, a few reports have suggested paravaginal repair (PVR) [36,37]; however, there have been conflicting results.

**Figure 1:**
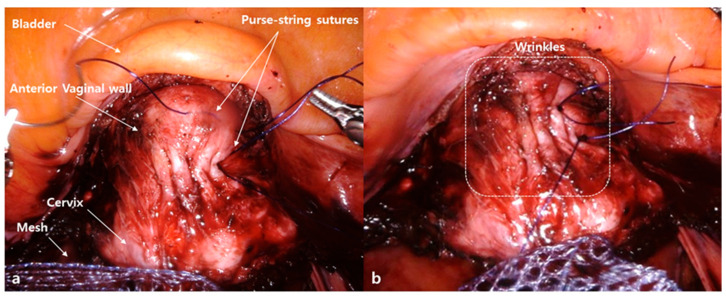
The wrinkle method. (**a**) Creation of two or three rows of purse-string sutures with delayed absorbable monofilament suture material (2-0 PDS; Ethicon, Johnson & Johnson, New Brunswick, NJ, USA) on the anterior vaginal wall. (**b**) Creation of wrinkles on the anterior vaginal wall and anchoring of the mesh on the wrinkle.

We propose a new “wrinkle method”, hypothesizing that this technique might decrease both anterior compartment recurrence and vaginal mesh erosion after SC. The wrinkle method consists of creating two or three rows of purse-string sutures with delayed absorbable monofilament suture material on the anterior vaginal wall and anchoring the mesh on the wrinkle (Figure 1).

**Figure 2:**
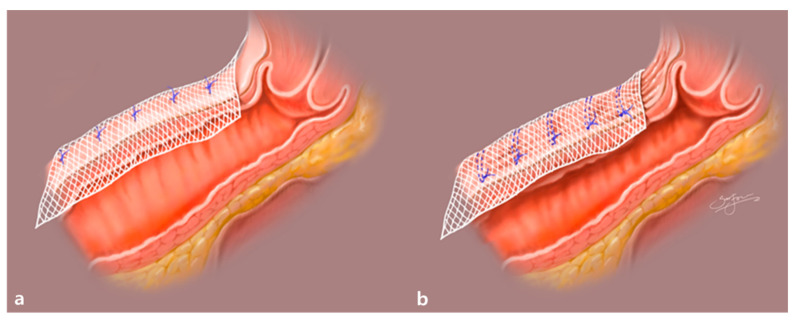
Comparison of the two surgical methods of mesh attachment during sacrocolpopexy. (**a**) The conventional, non-wrinkle method. (**b**) The wrinkle method. The wrinkle method can narrow the caliber of the vaginal canal, thicken the anterior vaginal wall, and broaden the anterior vaginal wall surface covered by the same amount of mesh.

Using this method, we can expect not only a decrease in the anterior vaginal wall width but also a narrowing of the vaginal canal caliber (Figure 2). Anchoring a mesh on the created wrinkles of the anterior vaginal wall also means that more anterior vaginal wall can be covered using the same amount of mesh. Noe G.K. et al., through the so-called defect-oriented strategy, suggested the limited use of mesh to decrease mesh-related complications [38]. Therefore, the wrinkle method might decrease the risk of recurrence of anterior compartment prolapse after SC. Furthermore, we can expect a lower risk of healing abnormality, and vaginal mesh erosion, resulting in a thicker anterior vaginal wall compared to the original anterior vaginal wall, as illustrated in Figure 2. On the other hand, the use of the wrinkle method may increase the total operating time (OT), with extra time spent on making two or three rows of purse-string sutures, and there may be a slight increase in blood loss during the creation of those sutures on the anterior vaginal wall.

The aim of this study was to suggest a new “wrinkle method” for intracorporeal anterior vaginal wall plication during RSC and to evaluate its feasibility. We also compared the anterior compartment prolapse recurrence rates and perioperative outcomes in patients who underwent RSC with or without the new wrinkle method.

## 2. Materials and Methods

### 2.1. Study Design and Patients

Patients with pelvic organ prolapse quantification (POP-Q) stage III–IV who underwent RSC with or without the new wrinkle method for symptomatic POP between 1 January 2018 and 30 June 2020, with a follow-up (FU) period of at least 3 months were included in this study. The wrinkle method was initially developed in March 2019 by one surgeon (Lee S.R.) and applied to cases operated on between April 2019 and June 2020.

We obtained the following data from each patient’s chart review: age, body mass index (BMI), detailed gynecologic, medical, and surgical histories, and POP-Q stage. We also collected the time of suturing for the creation of the wrinkle on the anterior vaginal wall by reviewing recorded surgical videos. Furthermore, we obtained the following surgery-related data: use of the wrinkle method, concomitant surgery, and total OT from skin incision to the completion of the skin closure including all the concomitant operations. Additionally, we collected data on perioperative outcomes, including estimated blood loss (EBL), any intra- or postoperative adverse events, length of hospital stay, postoperative fever within 48 h, and any complications related to the wrinkle method. Postoperative FU data for the recurrence of POP were collected as well. The diagnoses of POP, POP-Q staging, surgery, and postoperative FU were performed by a single urogynecologist (Lee S.R.). All surgical procedures were performed without modification with the same mesh and suture materials. The wrinkle method was applied in some cases but not in others. The physician was blinded to the use of wrinkle method during the postoperative FU examination; the data were collected by another gynecologist (Kim J.H.) and statistical analysis was performed by an independent statistician.

### 2.2. Surgical Procedures

The surgical steps were the same as the ones in our previous report [32,39]. In the case of multi-port RSC, a total of three incisions were made: a 2.5-cm intraumbilical and two 8-mm skin incisions; that is, the reduced port method was used in all cases. The da Vinci Si^®^ and da Vinci Xi^®^ systems (Intuitive Surgical, Sunnyvale, CA, USA) with central or side docking types were used. In the case of single-incision RSC, single-site RSC (SS-RSC) was performed using the da Vinci Si^®^ or da Vinci Xi^®^ system and single-port RSC (SP-RSC) was performed using the da Vinci SP system with central docking type. A 2.7-cm intraumbilical incision was made.

All surgeries were performed under general anesthesia, and all patients underwent standard operative care. The surgical materials, including suture materials and mesh, were almost the same in all cases. Non-absorbable suture materials (2-0 Ethibond; Ethicon, Johnson & Johnson, New Brunswick, NJ, USA or 2-0 Prolene; Ethicon Inc.) and absorbable suture material (2-0 PDS; Ethicon Inc.) were used as mesh anchoring sutures. Complete peritoneal closure was performed using absorbable barbed suture materials (2-0 Monofix PDO; Samyang, Daejeon, South Korea), 2-0 V-Loc (Covidien, Mansfield, MA, USA), or 1-0 Quill™ SRS bidirectional barbed suture (Angiotech Pharmaceuticals, Inc., Vancouver, BC, Canada). A Y-shaped, 2 cm × 18 cm size, partially absorbable macroporous polypropylene light-weight mesh (Seratex^®^ PA B2 type; Serag-Wiessner KG, Naila, Germany) was used in all cases. Most intra-corporeal procedures were the same in all cases and only differed in terms of whether the wrinkle method was used. Supracervical hysterectomy (SCH) with or without adnexectomy was performed, and the cervical stump was sutured with 1-0 V-Loc (Covidien) sutures using a continuous running suture technique. This step was omitted in the 20 cases of vault prolapse. Dissection of the avascular anterior vesicovaginal plane and posterior rectovaginal plane of approximately 6–7 cm in length was performed, and the mesh was sutured to fix approximately 5–6 cm of the anterior and posterior vaginal wall.

The wrinkle method was performed in the wrinkle group (*n* = 57), involving the creation of two or three rows of purse-string sutures with 2-0 PDS on the anterior vaginal wall and anchoring of the mesh on the wrinkle, as illustrated in Figure 1 and Figure 2. Dissection of the presacral area was performed to expose the ALL of the sacrum. Fixation of a Y-shaped mesh was performed using multiple discrete sutures (both absorbable and non-absorbable suture materials) on the anterior and posterior vaginal walls. Fixation of the cranial end of the Y-shaped mesh was performed using non-absorbable suture materials after adjusting the mesh tension. Complete closure of the peritoneum was performed with absorbable barbed suture materials to prevent mesh exposure, bowel adhesion, and bowel strangulation using the peritoneal tunneling method as previously described by Liu et al. [40]. Removal of the retrieved uterus and adnexae was performed using knife morcellation within an Endo-bag^®^ (Sejong Medical, Paju, Gyeonggi-do, South Korea).

### 2.3. Statistical Analysis

To compare continuous variables in the wrinkle and non-wrinkle groups, we used Student’s *t*-test. To compare the proportions of categorical variables between the two groups, we used the chi-squared test. The data were normally distributed (*p* > 0.05, Kolmogorov–Smirnov test). All computations were performed with R, a language and environment for statistical computation (R Foundation for Statistical Computing, Vienna, Austria) [41].

## 3. Results

### 3.1. Patient Baseline Characteristics

A total of 221 patients with POP-Q stage III–IV underwent RSC during the study period, and 123 patients were included in our analysis. Fifty-seven patients (reduced-port, *n* = 55; single incision, *n* = 2) underwent RSC with the wrinkle method (wrinkle group) and 66 patients (reduced-port, *n* = 20; single incision, *n* = 46) underwent RSC without the wrinkle method (non-wrinkle group). The FU period was 24.03 ± 4.26 months (median, 23.5 months (range, 14–31 months)) for the non-wrinkle group and 9.13 ± 4.61 months (median, 10 months (range, 3–16 months)) for the wrinkle group. The mean age of patients was significantly higher in the non-wrinkle group (59.05 ± 9.59 years and 63.29 ± 10.97 years in the wrinkle and non-wrinkle groups, respectively). The mean BMI was comparable between the two groups. There were no differences in the proportion of menopausal patients, history of previous pelvic surgery, vault prolapse, or distribution of preoperative POP-Q stages between the two groups (Table 1).

### 3.2. Comparison of Surgical Outcomes between the Wrinkle and Non-Wrinkle Groups

The mean total OT was 109.71 ± 26.45 min in the wrinkle group and 125.35 ± 28.24 min in the non-wrinkle group, and OT was significantly shorter in the wrinkle group. In terms of concomitant procedures rates, there was no difference in the rate of SCH or application of trans-obturator tension free vaginal tape (TOT) between the two groups. However, the rates of adnexectomy and adhesiolysis were significantly higher in the non-wrinkle group, whereas the rate of posterior colpoperineorrhaphy was significantly higher in the wrinkle group. The mean EBL was comparable and below 75.0 mL in both groups (64.42 ± 50.3 mL vs. 70.08 ± 67.8 mL in the wrinkle and non-wrinkle groups, respectively) (Table 2).

### 3.3. Comparison of Intraoperative and Postoperative Adverse Surgical Outcomes

In terms of operative adverse events, no patient in either group required transfusion or conversion to laparotomy or to multiport RSC of single-incision RSC. There were no cases of bowel obstruction, deep vein thrombosis, pulmonary embolism, or cardiac, respiratory, renal, or neurological complications. There were four cases of bladder injury (cystotomy) during surgery, two cases in the wrinkle group and two cases in the non-wrinkle group. This complication was resolved using intraoperative primary repair of the cystotomy site, extended urinary drainage with an indwelling urethral Foley catheter for 7 days, and removal of the Foley catheter after confirmation of no extravesical leakage of dye on cystography. There was no difference in the rate of complications between the two groups.

At the postoperative 4-week FU, all cases were at POP-Q stage 0–I, except two non-wrinkle group patients (2/66, 3.03%) with anterior compartment recurrence that was at POP-Q stage II. There was one case of umbilical incisional hernia in the non-wrinkle group (1/66, 1.51%) in an 86-year-old obese (BMI, 28.76 kg/m^2^) woman. This patient developed umbilical incisional hernia during the 3-month FU, and POP recurred. Although primary umbilical facial closure and anterior colporrhaphy had been performed concomitantly, 6 months later, symptomatic POP-Q stage II anterior compartment recurrence was noted again; however, the patient did not desire further surgical treatment. There was one other case of POP recurrence in the non-wrinkle group; however, the patient had no symptoms, and therefore no further treatment was administered.

There was one patient (1/57, 1.75%) with a retroperitoneal abscess in the wrinkle group preceded by a large hematoma that formed following bleeding during retroperitoneal dissection. The patient complained of postoperative ileus, and fever developed during the 5-day postoperative period. The abscess was drained using a double J catheter and intravenous antibiotics were administered. The mesh was not removed because there was no sign of mesh infection, and the patient recovered well without recurrence of POP. There was one case (1/66, 1.51%) of de novo stress urinary incontinence in the non-wrinkle group, which was resolved after anti-incontinence surgery (using TOT) performed 6 months postoperatively. There were two cases (2/66, 3.03%) of posterior vaginal wall mesh erosion of approximately 1 cm in size in the non-wrinkle group (Table 3). The exposed mesh and surrounding vaginal wall were removed under local anesthesia. Primary vaginal wall closure with absorbable suture material (2-0 Vicryl; Ethicon Inc.) was performed, and 2 mg/day oral estradiol was administered along with a 0.03 mg/day estriol vaginal tablet for 4 weeks. However, mesh erosion recurred in one patient and additional surgical management was planned. There were no cases of mesh erosion on the anterior vaginal wall or adjacent organs, such as the bladder or rectum, in either group.

## 4. Discussion

### 4.1. Feasibility of the Wrinkle Method during RSC

We designed this study to investigate the feasibility of the new wrinkle method during RSC and to evaluate the perioperative outcomes. This method may broaden the anterior vaginal wall surface covered by mesh and may also narrow the caliber of the vaginal canal. Furthermore, the wrinkle method may decrease the rate of mesh erosion by thickening the anterior vaginal wall below the mesh. A similar hypothesis was proposed by Noe G.K. et al. They have been performing the laparoscopic anterior and posterior native tissue repair since 2015, and they published an article demonstrating the safety of the new surgical method for the treatment of midline cystocele and rectocele [42]. The authors compressed and narrowed the endopelvic fascia and rectovaginal fascia by absorbable woven sutures instead of plication. The method enabled the vaginal approach switch to be performed entirely laparoscopically and used the same dissection plane with SC. Suturing the fascia can replace the insertion of the Y-shaped mesh, thus limiting the risk of mesh-related complications [42,43].

Similarly, we hypothesized that the wrinkle method would decrease the recurrence of POP and vaginal wall erosion after RSC; however, the short follow-up period of our retrospective study precludes any conclusion about the effect on the risk of vaginal mesh erosion and POP recurrence. Furthermore, in the current study, erosion was only noted in the non-wrinkle group, and the difference was statistically insignificant. Moreover, erosion was noted only in the posterior vaginal wall, not in the anterior vaginal wall where we created the wrinkles. Therefore, we could not conclude on the effect of the wrinkle method in terms of decreasing the rate of the vaginal wall erosion. Long-term FU data and randomized controlled trials with large numbers of patients, calculating the sample size with high power, are required to determine this effect.

### 4.2. Previous Reports on POP Recurrence and Mesh Erosion Rates after SC

Concerning objective POP recurrence after SC, van Zanten et al. [34] published a bi-center prospective cohort study of 305 patients who underwent RSC or SCH with sacrocervicopexy (RSHS) in 2019. Anatomical success of the apical compartment occurred in 91% (RSC) and in 99% (RSHS) of the women. Most recurrences were in the anterior compartment (15.7% RSC; 22.9% RSHS). Unger et al. [44] reported a retrospective cohort study of 406 women who underwent RSC or LSC during 2006–2012. At the 6-month FU, 10 women were found to have undergone reoperation for POP recurrence. Most (80%, 8/10) reoperations for POP were for symptomatic rectoceles and not for anterior or apical prolapse, despite the fact that concomitant rectocele repair was performed in the LSC (48.7%) and RSC (33.1%) groups. A meta-analysis conducted in 2016, including two randomized controlled trials (RCTs) as well as one prospective cohort and six retrospective cohorts, concluded that RSC was associated with higher recurrent POP and reoperation than LSC [45]. Recently, Thomas et al. [46] compared open SC, LSC, and RSC. The authors reported that within the year after surgery, the overall objective prevalence of POP recurrence was 15% (open 11.7%, robotic 21.1%, laparoscopic 13.8%), and the prevalence of mesh exposure was 5.3% (open 7.7%, robotic 3.6%, laparoscopic 4.9%). At 6.5 (1.6–13.4) years, the overall patient-reported prevalence of POP recurrence was 9.2% (open 6.3%, robotic 12.5%, laparoscopic 8.5%), and the prevalence of mesh exposure was 6.9% (open 6.0%, robotic 3.9%, laparoscopic 8.6%). The reason for the low POP recurrence in both groups may have been the relatively short FU period (range, 6 to 36 months). The rates of recurrent POP symptoms were 13.1%; however, only 2.5% of patients underwent reoperation.

### 4.3. Previous Reports on Prevention of Recurrent POP after SC

Attempts to prevent recurrent POP after SC have been reported. The reduction in recurrent POP of the anterior compartment is challenging because the anterior compartment is most commonly involved after SC [28,29,30]. Cronjé et al. [47] published a case report of performing open SC to a patient with POP-Q stage IV by extending the length of the mesh along the vaginal walls to reduce recurrent vaginal prolapse.

In terms of PVR, it was theorized that a paravaginal defect may cause a lateral cystocele, and, therefore, concomitant PVR during SC was considered to be helpful in anterior POP recurrence. The effect of concomitant PVR to decrease a recurrent cystocele was initially reported in open SC by Shippey et al. in 2010 [37]. The authors compared open SC with PVR (*n* = 62) or without PVR (*n* = 108) at the 16.0- and 12.9-month FUs, respectively. However, there was only a trend toward improved clinical outcomes, which was not statistically significant. Hoke et al. [36] also compared the anatomic outcome in RSC with PVR (*n* = 21) or without PVR (*n* = 102) at the 3-month FU. However, concomitant PVR performed during RSC also failed to provide significant objective and subjective improvement. The authors stated that this result may be attributed to the fact that patients undergoing RSC with PVR usually had worse baseline prolapse, and a prospective RCT will be needed to conclude on the effect of PVR on the decrease in POP recurrence after RSC.

In our study, no case of mesh erosion on the anterior vaginal wall was noted, and all cases (1.63%, 2/123) involved the posterior vaginal wall. A thin or injured posterior vaginal wall during electrocauterization for the bleeding on the posterior vaginal wall during rectovaginal wall dissection may have predisposed patients to posterior vaginal erosion. The reason for the lack of cases of mesh erosion in the wrinkle group may be the shorter FU periods in the wrinkle group. After SC, both mesh erosion and POP recurrence rates are known to increase over long-term FUs, and the probability of mesh erosion was reported to reach 10.5% (95% CI, 6.8% to 16.1%) at the 7-year FU in an RCT for open SC [48]. Unger et al. [44], reported mesh erosion at 2.7%, and it was not statistically different between LSC and RSC, nor between patients who underwent concomitant hysterectomy and those who did not. A meta-analysis reported two cases (1.25%, 2/160) of mesh-related reoperation performed with LSC and zero cases (0/54) with RSC [45]. A recent study comparing the long-term outcomes after LSC and RSC reported mesh-related reoperations performed in 0.93% of patients (2/214, one case for vaginal mesh erosion and one case for the infectious spondylodiscitis) and all cases were noted with LSC [49]. However, whether the vaginal mesh erosion occurred in the anterior or posterior vaginal wall was not described in detail in most reports.

### 4.4. Comparison of Total OT in the Wrinkle and Non-Wrinkle Groups

The total OT was significantly shorter in the wrinkle group without differences in EBL or an increase in perioperative complications. We had expected that when reviewing the full surgical videos, we would find that an additional 4–6 min of surgical time would be required for the wrinkle method and ultimately a longer total OT. However, this was not the case, and the reason for the shorter total OT in the wrinkle group may be attributable to improvements in surgical proficiency over the study period. The learning curve for robotic surgery is known to be steeper than that of laparoscopic surgery. In terms of RSC, initial reports have suggested that RSC is associated with longer surgery duration than LSC [50,51]. However, the bias of inexperience with robot assistance was not considered in these initial reports on RSC. Illiano et al. [52] indicated that the OTs for RSC were longer than those for LSC, but they did not consider the robot docking time. Seror et al. [53] evaluated the pure RSC OT and LSC OT, excluding the time for docking of the robot, and found that the pure RSC OT was 95 min less than the LSC OT, demonstrating the superiority of RSC in terms of OT. Van Zanten et al. [34] reported that the difference in OT between RSC and LSC was only 13.6 min for experienced surgeons.

Additionally, the lower rate of concomitant adnexectomy and adhesiolysis and the higher rate of posterior colpoperineorrhaphy in the wrinkle group than in the non-wrinkle group may also result in a shorter total OT. Therefore, a well-designed randomized control study should be conducted to reach further conclusions.

### 4.5. Strengths and Limitations

This study had several strengths. First, to the best of our knowledge, this is the first study to suggest a new wrinkle method and compare RSC with and without the use of the wrinkle method. Second, the relatively short study period of 30 months meant that surgical procedures were relatively consistent. Third, this is the experience of a single surgeon, and this minimized the influence of variations in surgical experience and proficiency across different surgeons. Fourth, we used a validated POP quantification system both pre-and postoperatively. Fifth, the wrinkle method was not time-consuming, was easy to perform, was applicable to abdominal SC and LSC, and did not require additional training or instruments. Finally, none of the cases required additional skin incision or mini-laparotomy.

However, this study has some limitations that should be considered. First, this was a single-center retrospective study and not a multicenter RCT with a large number of patients and calculation of the sample size with high power. Second, both groups comprised a relatively small number of cases, and the relatively short-term FU data precluded us from concluding on the long-term efficacy and safety of the wrinkle method. Third, the wrinkle method was applied to cases operated after April 2019 and the FU period was significantly different between the two groups. This is the main reason we cannot conclude on the effect of wrinkle on recurrence and mesh erosion, both affected by the FU periods. Fourth, we did not evaluate the sexual function of patients and their partners following RSC using a validated questionnaire, which can help reveal the effect of the wrinkle method. Narrowing the vaginal caliber and creating wrinkles may have positive effects on sexual function; however, the thickening of the vaginal wall may negatively affect the patient’s sexual sensation. Finally, as a single surgeon performed the surgery in all cases, we cannot exclude the possibility of selection biases.

## 5. Conclusions

In conclusion, performing the proposed wrinkle method during RSC is simple and not time-consuming. It may decrease anterior POP and vaginal erosion; however, long-term RCTs are essential to confirm our findings.

## Figures and Tables

**Table 1:** Patient baseline characteristics.

Characteristics	Wrinkle (*n* = 57)	Non-Wrinkle (*n* = 66)	*p* Value
Age (years, mean ± SD)	59.05 ± 9.59	63.29 ± 10.97	0.02
BMI (kg/m^2^, mean ± SD)	24.02 ± 2.56	23.55 ± 2.97	0.38
Menopause, *n* (%)	43 (75.4)	59 (89.4)	0.07
Vaginal delivery, (median (range))	2 (0–8)	2 (0–9)	0.12
Previous pelvic surgery, *n* (%)	17 (29.8)	30 (45.5)	0.11
Vault prolapse, *n* (%)	9 (15.8)	11 (16.7)	1
POP-Q stage, *n* (%)			1
Stage III	43 (75.4)	53 (80.3)	
Stage IV	14 (24.6)	13 (19.7)	

SD, standard deviation; BMI, body mass index; POP-Q, pelvic organ prolapse quantification.

**Table 2:** Comparison of operative outcomes.

	Wrinkle (*n* = 57)	Non-Wrinkle (*n* = 66)	*p* Value
Total OT, (min, mean ± SD)	109.71 ± 26.45	125.35 ± 28.24	0.003
Concomitant surgery, *n* (%)			0.26
Supracervical hysterectomy	49 (86)	55 (83.3)	0.88
Adnexectomy	14 (71.9)	59 (89.4)	0.02
Adhesiolysis	2 (3.5)	21 (31.8)	<0.001
Posterior colpoperineorrhaphy	15 (26.3)	6 (9.09)	0.02
TOT	17 (29.8)	12 (18.2)	0.19
Estimated blood loss (mL, mean ± SD)	64.42 ± 50.3	70.08 ± 67.8	0.61

OT, operation time from skin incision to the completion of the skin closure including all the concomitant operations; TOT, trans-obturator tension-free vaginal tape; SD, standard deviation.

**Table 3:** Comparison of intraoperative and postoperative adverse events.

	Wrinkle (*n* = 57)	Non-Wrinkle (*n* = 66)	*p* Value
Intraoperative AEs, *n* (%)			
Bladder injury	2 (3.5)	2 (3.03)	1
Bowel injury	0	0	1
Postoperative AEs, *n* (%)			
Bowel obstruction	0	0	1
Urinary tract infection	0	0	1
Retroperitoneal abscess and fever	1 (1.75)	0	1
Umbilical incisional hernia	0	1 (1.51)	0.98
De novo stress urinary incontinence	0	1 (1.51)	0.98
Posterior vaginal wall—mesh erosion	0	2 (3.03)	0.97
Recurrence of anterior compartment POP	0	2 (3.03)	0.97

AEs, adverse events; TOT, trans-obturator tension-free vaginal tape; SD, standard deviation; POP, pelvic organ prolapse.

## Data Availability

The excel data used to support the findings of this study were supplied by Sa Ra Lee under license, and requests for access to these data should be made to S.R.L. leesr@amc.seoul.kr.
